# En bloc resection of huge primary tumors with epidural involvement in the mobile spine using the “rotation–reversion” technique: Feasibility, safety, and clinical outcome of 11 cases

**DOI:** 10.3389/fonc.2022.1031708

**Published:** 2022-12-01

**Authors:** Ming Lu, Zhongxin Zhou, Wei Chen, Zixiong Lei, Shuangwu Dai, Changhe Hou, Shaohua Du, Qinglin Jin, Dadi Jin, Stefano Boriani, Haomiao Li

**Affiliations:** ^1^ Department of Musculoskeletal Oncology, Center for Orthopaedic Surgery, The Third Affiliated Hospital of Southern Medical University, Guangzhou, China; ^2^ Department of Interventional Vascular Surgery, The Third Affiliated Hospital of Southern Medical University, Guangzhou, China; ^3^ GSpine4 Spine Surgery Division, Istituto Di Ricovero E Cura A Cacattere Scientifico (IRCCS), Istituto Ortopedico Galeazzi, Milan, Italy

**Keywords:** en bloc resection, spinal tumor, epidural involvement, huge mass, safe margin, rotation-reversion technique

## Abstract

**Background:**

En bloc resection of spinal tumors provides better local control and survival outcomes than intralesional resection. Safe margins during en bloc resection of primary spinal tumors with epidural involvement are required for improved outcomes. The present study describes a “rotation–reversion” technique that has been used for en bloc resection of huge primary tumors in the mobile spine with epidural involvement and reported the clinical outcomes in these patients.

**Methods:**

All patients with primary spinal tumors who were treated with the rotation–reversion technique at our institution between 2015 and 2021 were evaluated retrospectively. Of the patients identified, those with both huge extraosseous soft-tissue masses and epidural involvement were selected for a case review. Clinical and radiological characteristics, pathologic findings, operative procedures, complications, and oncological and functional outcomes of these patients were reviewed.

**Results:**

Of the 86 patients identified with primary spinal tumors who underwent en bloc resection using the rotation–reversion technique between 2015 and 2021, 11 had huge extraosseous soft-tissue masses with epidural involvement in the mobile spine. The average maximum size of these 11 tumors was 8.1 × 7.5 × 9.7 cm. Median follow-up time was 28.1 months, mean operation time was 849.1 min (range 465–1,340 min), and mean blood loss was 6,972.7 ml (range 2,500–17,700 ml), with 10 (91%) of the 11 patients experiencing perioperative complications. The negative margin rate was 91%, with only one patient (9%) experiencing local recurrence. Ten patients were able to walk normally or with a crutch at the last follow-up, whereas one was completely paralyzed preoperatively.

**Conclusion:**

The rotation–reversion technique is an effective procedure for the en bloc resection of huge primary spinal tumors, with the extension of invasion in selected patients including not only the vertebral body but also the pedicle and part of the posterior arch.

## 1 Introduction

Primary tumors occurring in the mobile spine are rare, with an estimated incidence of 2.5–8.5 per million people per year, accounting for 4%–13% of all primary bone tumors (1). Primary spinal tumors can occur in any region of the spine, most commonly in the thoracic and lumbar spine.

En bloc resection of tumors with oncologically appropriate margins is the optimal treatment for primary malignant tumors of the mobile spine (2–5). Obstacles to en bloc resection of spinal tumors include regional anatomical limitations and large tumor volumes (6–8). Due to the lack of surrounding anatomical barriers over the surface of the vertebral body, bone tumors that break through the cortical bone and longitudinal ligament can easily grow into huge masses outside the spinal column (9). Factors associated with large tumor sizes include the long duration of symptoms before seeking medical care (sometimes from months to years) and recurrence after non-standard operations (e.g., curettage or intralesional resection). Serious damage to surrounding neurovascular structures is a frequent cause of morbidity associated with en bloc resection of large spinal tumors (10).

Tumor epidural extension is commonly seen in spinal tumors, as is tumor extension that includes not only the vertebral body but also the pedicle and part of the posterior arch (11, 12). The inability to visualize dorsal structures of the vertebral column has limited the ability to achieve negative tumor margins during the surgical management of spinal tumors with epidural involvement. Recurrences along the dura are frequently due to inadvertent intraoperative contamination (12).

Although several studies have described the en bloc resection of multi-level spinal tumors (13–16), less is known about methods that achieve safe margins in huge spinal tumors with epidural involvement, in which extensions include not only the vertebral body but also the pedicle and part of the posterior arch. The present study retrospectively analyzed a group of patients with huge primary tumors in the mobile spine and epidural involvement who underwent en bloc resection using the “rotation–reversion” technique. The details of this technique are described, and its safety and feasibility are determined by analyzing oncological and clinical outcomes.

## 2 Patients and methods

### 2.1 Study participants

The medical records and follow-up results of patients with primary spinal tumors who underwent en bloc tumor resection using the rotation–reversion technique at our institution between 2015 and 2021 were retrospectively reviewed. Imaging and medical records were reviewed manually, and patients who met the following inclusion and exclusion criteria were selected for this study.

Patients were included if histological examination of preoperative core needle biopsy samples confirmed a diagnosis of primary tumor of the mobile spine; if extension and location of the tumor, as classified by the Weinstein–Boriani–Biagini (WBB) staging system (17), involved both extraosseous soft tissues (layer A) and the extradural layer (layer D), with the extension of intraosseous invasion including not only the vertebral body but also the pedicle and part of the posterior arch; and if they were followed up postoperatively for >6 months. Patients were excluded if they had tumor extension with intradural involvement (layer E), if their clinical or imaging data were incomplete, or if they were lost to follow-up.

Factors recorded for each patient included their clinical and radiological characteristics, pathology results, operative procedure, complications, and oncological and functional outcomes. All patients provided written informed consent, and the study protocol was reviewed and approved by the ethics committee of the Third Affiliated Hospital of Southern Medical University.

### 2.2 Evaluation and decision making

Radiographs, CT, MRI of the spine, and PET-CT were performed on all patients. Histological diagnosis was achieved based on core needle biopsy preoperatively. The treatment strategy for each patient was determined by a multidisciplinary collaboration performed by the same team consisting of surgeons, radiation oncologists, medical oncologists, radiologists, and pathologists. Once the surgical plan was determined, an elaborated design of the surgical procedures and the potential complications were discussed and planned carefully by our multidisciplinary collaboration team before the operation. Selective arterial embolization (SAE) was performed 1 day prior to the surgery in all cases. Intraoperative neuromonitoring was only used for the thoracic spine with more than three levels involved or with the cervical spine involved. All surgeries were performed by the same surgeon (HL).

### 2.3 Surgical technique

The surgical procedure was designed to provide adequate surgical margins as described (17, 18). The surgical approach was based on the location and extent of tumor invasion and the affected spinal level. Surgical approaches included three cases with a one-stage posterior approach and eight with the combined anterior and posterior approach.

#### 2.3.1 One-stage posterior approach

**Figure 1:**
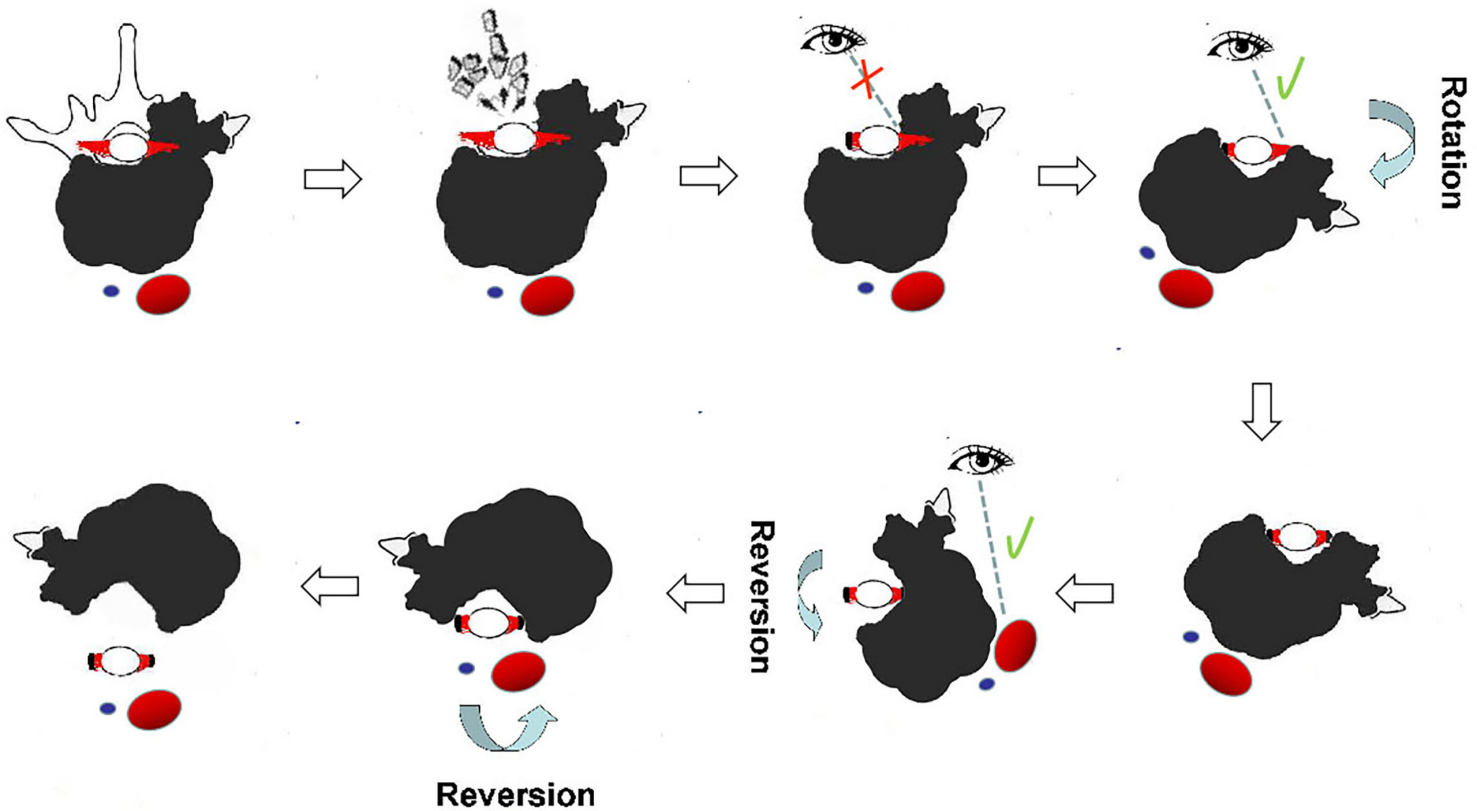
Illustration of en bloc resection using the rotation–reversion technique through a single posterior approach in the thoracic spine. A safe window was opened at the posterior arch, and nerve roots on the unaffected side were sectioned. The dura was separated from the lesion, and the nerve roots on the side covered by the tumor were sectioned under direct visualization by the rotation technique. The great vessels and surrounding structures at the ventral side of the tumor-invaded vertebrae were bluntly dissected under direct visualization using the reversion technique from the posterior approach. Reversal of the entire mass was continued until it was completely removed posteriorly.

Patients who underwent en bloc resection using a one-stage posterior approach were placed in the prone position (**Figure 1**). Transpedicular screws were inserted into the segments adjacent to the lesion, and all structures surrounding the part of the posterior arch of the diseased vertebra(e) were separated according to the designed margin. The great vessels and structures surrounding the ventral side of the vertebra(e) were carefully dissected along the anterior margin using a spatula and the surgeon’s fingers. If the great vessels and surrounding structures could not be bluntly dissected, due to large tumor size or severe adhesions, only the planned osteotomy levels at the caudad and cephalad of the vertebra(e) invaded by tumor were bluntly dissected, and two wire saws were installed. The tumor was subsequently resected en bloc using the rotation–reversion technique. Briefly, after excision of the normal healthy bony structure (lamina and pedicle), a safe window was opened at the posterior arch of the vertebra(e) invaded by the tumor, allowing entry into the spinal canal, and nerve roots on the unaffected side were sectioned. Because the nerve roots on the side of the tumor invasion and part of the dural mater were covered by the tumor, they could not be exposed directly due to the lamina and pedicle invasion, making it very difficult to obtain safe margins along the dura. Osteotomy was performed at the caudad and cephalad discs or the vertebral bodies of the vertebra(e) invaded by the tumor. The specimen was rotated around the longitudinal axis to the side of the lesion, enabling direct visualization of the dorsal structures of the spinal column. This allowed the dura to be separated from the lesion and the nerve roots to be sectioned without violating the tumor pseudocapsule at the lamina of the side covered by the tumor. With the use of the reversion technique, the great vessels and surrounding structures at the ventral side of the tumor-invaded vertebra(e) could be reversibly rotated. The vertebra(e) was thereafter somewhat on the longitudinal axis, allowing blunt dissection to be carefully performed under direct visualization from the posterior approach, thereby avoiding damage without violating the tumor pseudocapsule. Finally, the entire mass was removed posteriorly using the reversing maneuver. The anterior defect was reconstructed, followed by fixation of the pedicle screws using a posterior approach.

#### 2.3.2 Combined approach

**Figure 2:**
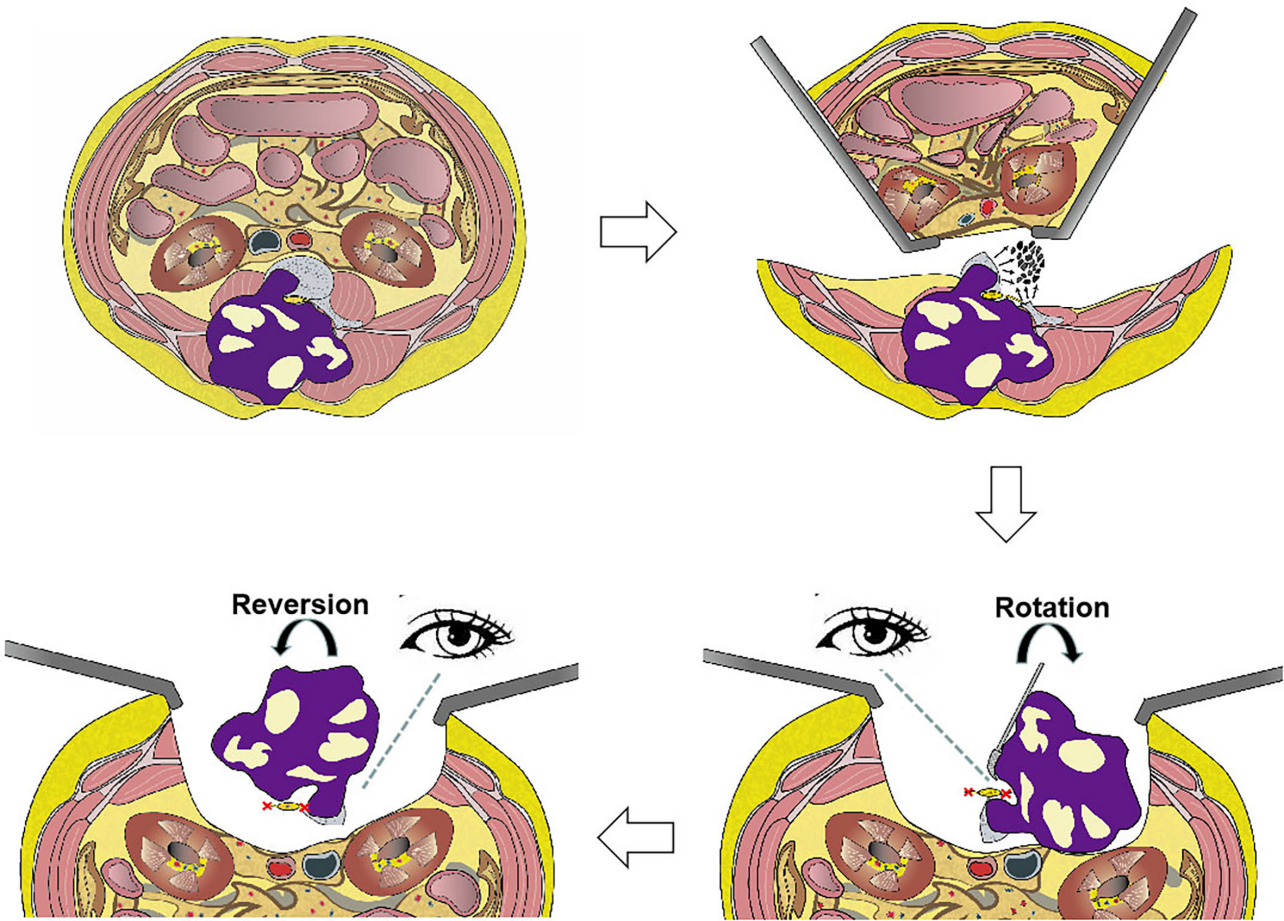
Illustration of en bloc resection using the rotation–reversion technique using a combined anterior and posterior approach in the lumbar spine. The retroperitoneal space was entered through a bilateral anterior pararectus approach, and the aorta and inferior vena cava were separated from the diseased vertebral body. The left hemivertebral body and pedicle of the diseased vertebrae were piecemeal removed as a safe window to enter the spinal canal. The mass in the bilateral paravertebral soft tissue that was not infiltrated by the tumor was separated using a posterior approach. The dura was separated from the mass, and the bilateral never roots of the diseased vertebrae were sectioned under direct visualization by the rotation technique. Finally, the entire mass was removed posteriorly using the reversion technique.

Some patients underwent combined procedures, as described (**Figure 2**). First, the tumor was released anteriorly from surrounding neurovascular structures all along the anterior margins. If sagittal en bloc resection was planned, osteotomy was performed using an ultrasonic osteotome, with an anterior approach performed from the healthy side of the spine anteroposteriorly along the sagittal plane of the vertebra(e). Second, the patient was placed in a prone position for posterior en bloc resection. Transpedicular screws were inserted into the upper and lower segments, and all the structures surrounding the diseased vertebra(e) along the posterior margin were released. A temporary rod was fixed to stabilize the spine and avoid spinal cord injuries during osteotomy. Finally, the lesion was removed en bloc using the rotation–reversion technique described above.

### 2.4 Follow-up

All patients were evaluated radiographically and by CT scans and MRI immediately after surgery and during follow-up. Oncologic outcomes were evaluated, including monitoring the sites for residual lesions, local recurrence, and signs of metastases. Functional results evaluated included neurologic function, hardware failure, and interbody fusion.

## 3 Results

### 3.1 Patient characteristics

**Figure 3:**
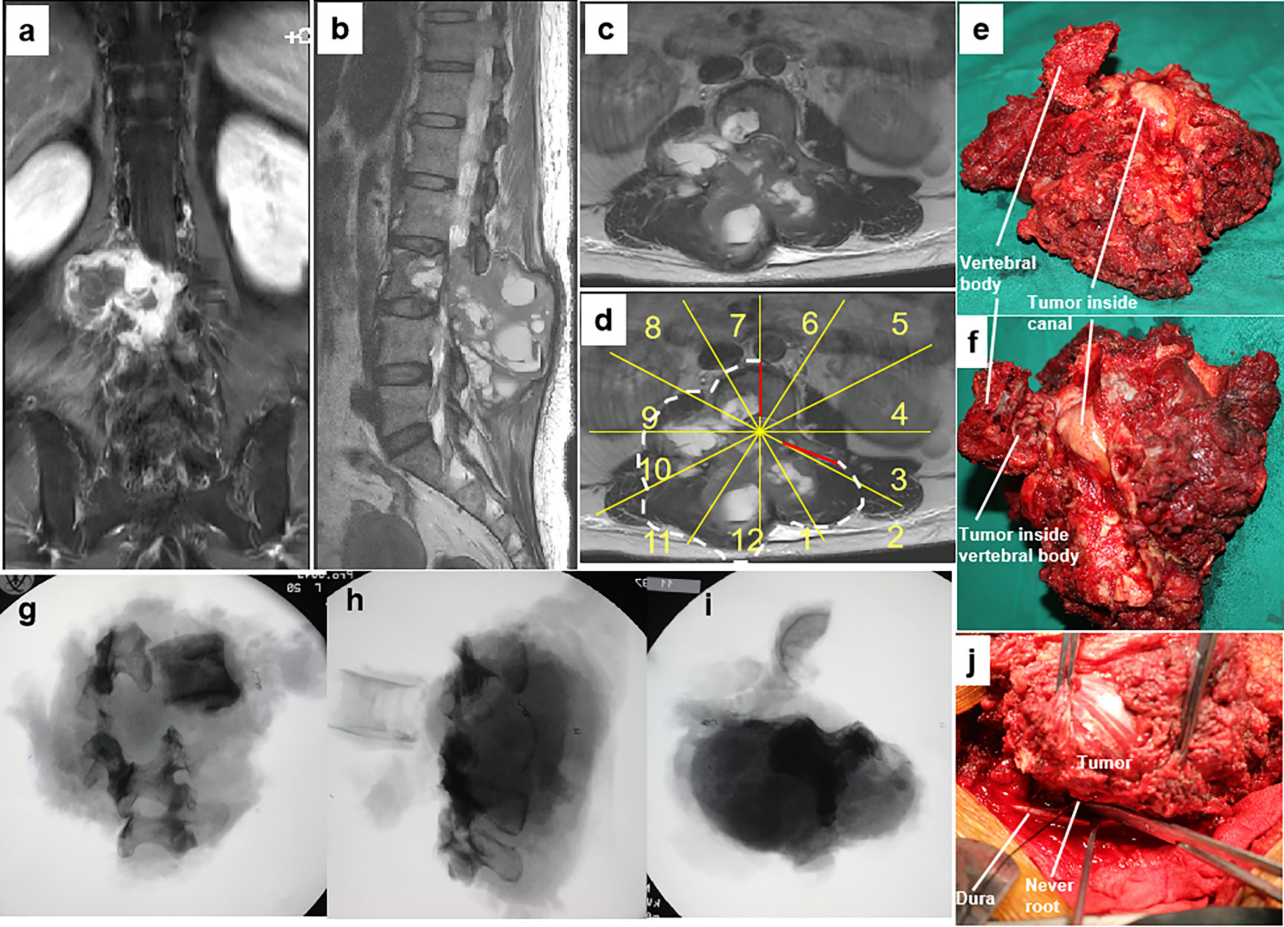
A 46-year-old woman with a malignant neurilemmoma at L3 with a huge mass in the posterior elements **(A–C)**. En bloc resection through a combined anterior and posterior approach was designed based on WBB classification along the margins, as highlighted by white dotted line (sectors 7–2, D, WBB) **(D)**. The dura was separated from the mass, and bilateral nerve roots of L3 were sectioned under direct visualization using the rotation technique **(J)**. Photographs of the gross specimen **(E, F)** showing that the epidural extension of the tumor was fully contained by the ligamentum flavum. Radiographs of the specimen **(G–I)** showing the margins of en bloc resection. WBB, Weinstein–Boriani–Biagini.

**Figure 4:**
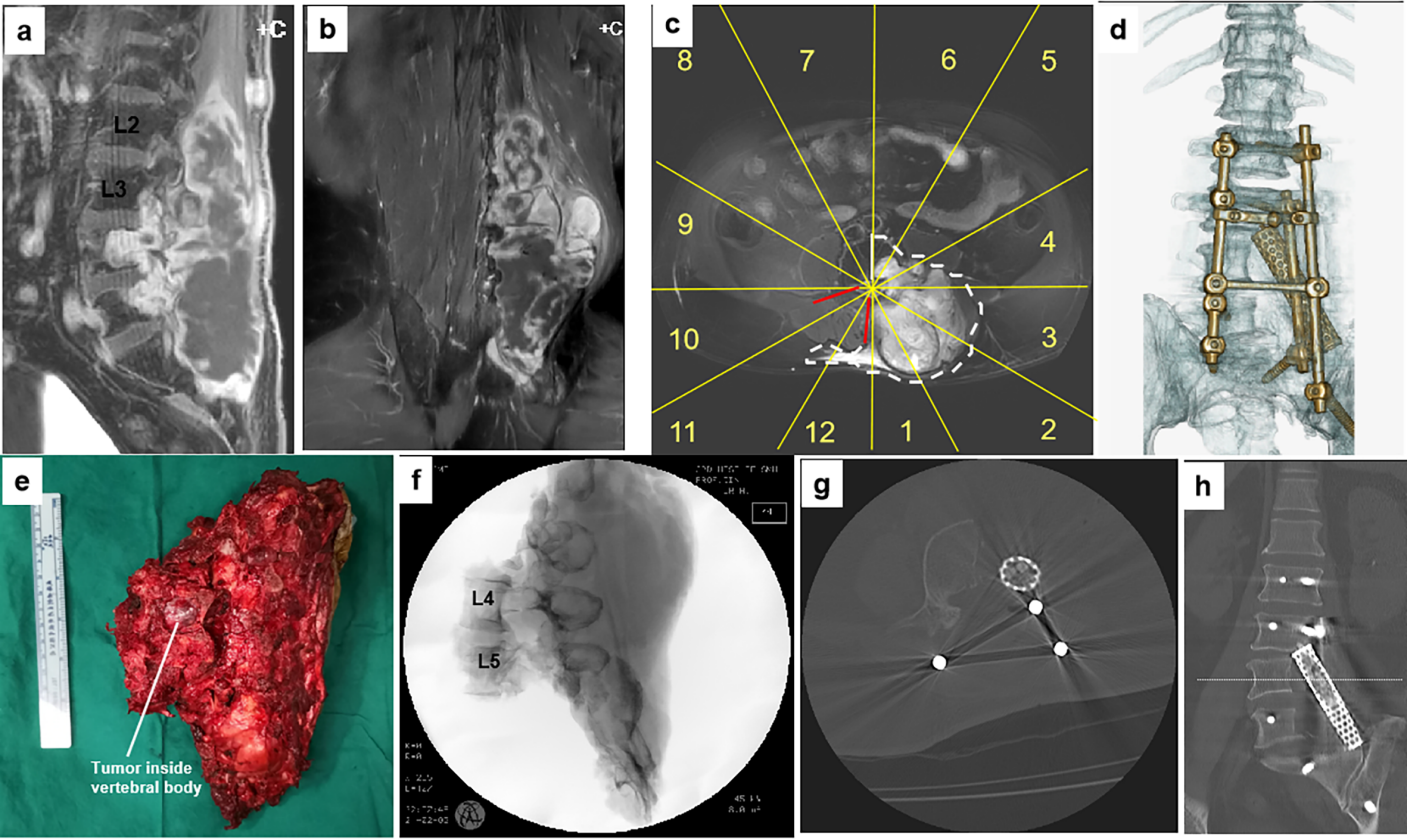
A 61-year-old man with a malignant neurilemmoma at L4–5. Preoperative MR images **(A–C)** show the tumor invading the left side of the L4 and L5 vertebrae sectors 1–6, **(D)**, WBB with a huge mass in the posterior elements (from L2 to S3). The patient underwent a two-stage surgery, consisting of anterior release followed by posterior sagittal en bloc resection with instrumentation. A photo of the gross specimen **(E)** and postoperative CT scan imaging **(F–H)** showing the margins on sagittal scans of en bloc resection. Postoperative CT scan reconstruction shows structural reconstruction with instrumentation after sagittal resection **(D)**. WBB, Weinstein–Boriani–Biagini.

**Figure 5:**
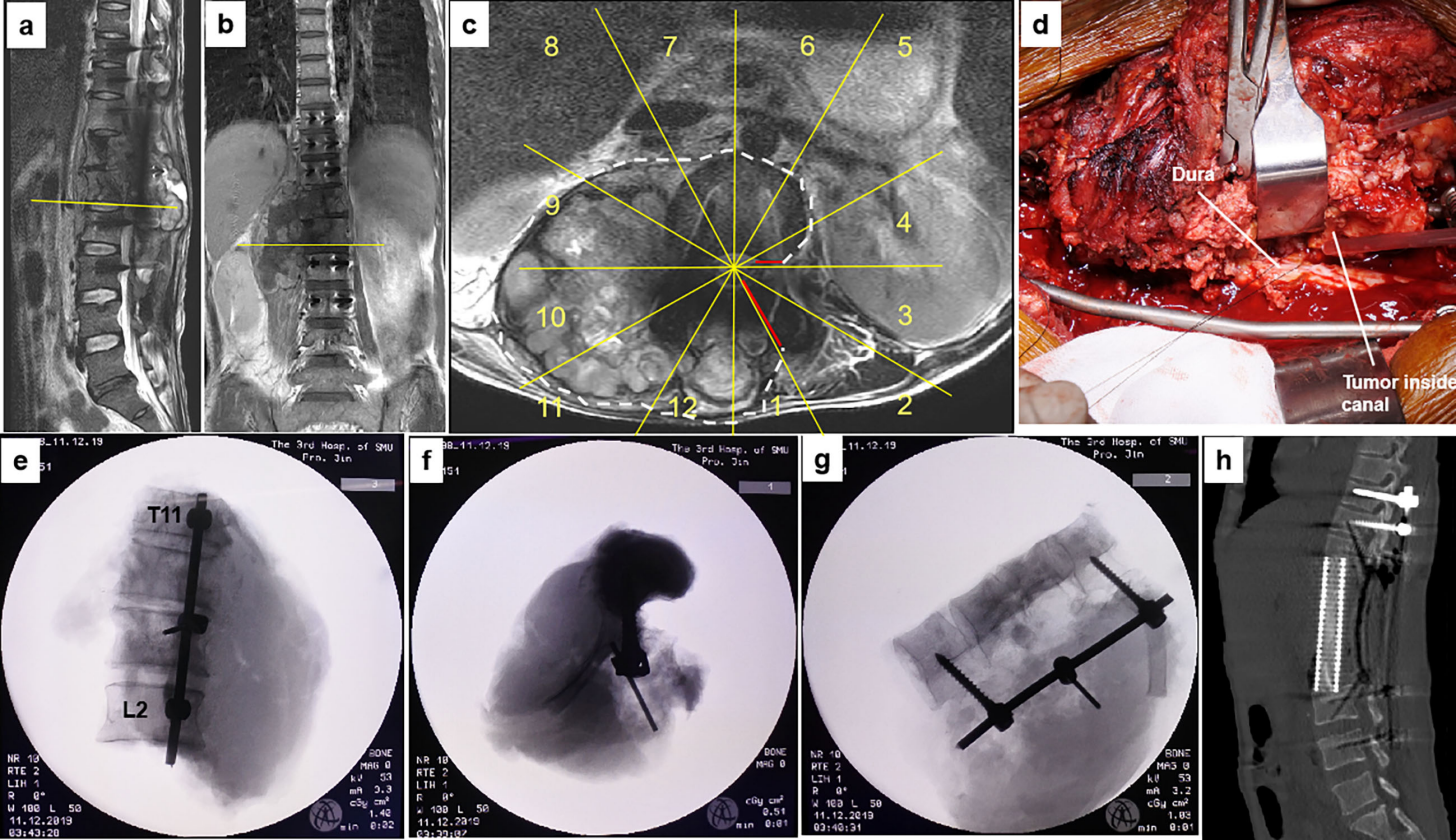
A 40-year-old man with osteosarcoma at T11-L2. This patient experienced a local tumor recurrence after two rounds of intralesional excision surgeries combined with radiotherapy in another hospital. MR images **(A–C)** show tumor recurrence along the right side of T11 to L2 paravertebral with epidural involvement (sectors 4–1, D, WBB). Based on the WBB classification, a four-level (T11-L2) en bloc spondylectomy was performed using the rotation–reversion technique after three courses of chemotherapy. However, due to intracanal tumor contamination caused by the initial operations, the margin along the dura was considered positive **(D)**. Radiographs of the specimen **(E–G)** and postoperative CT scans showing structural reconstruction with instrumentation after tumor resection **(H)**. WBB, Weinstein–Boriani–Biagini.

**Figure 6:**
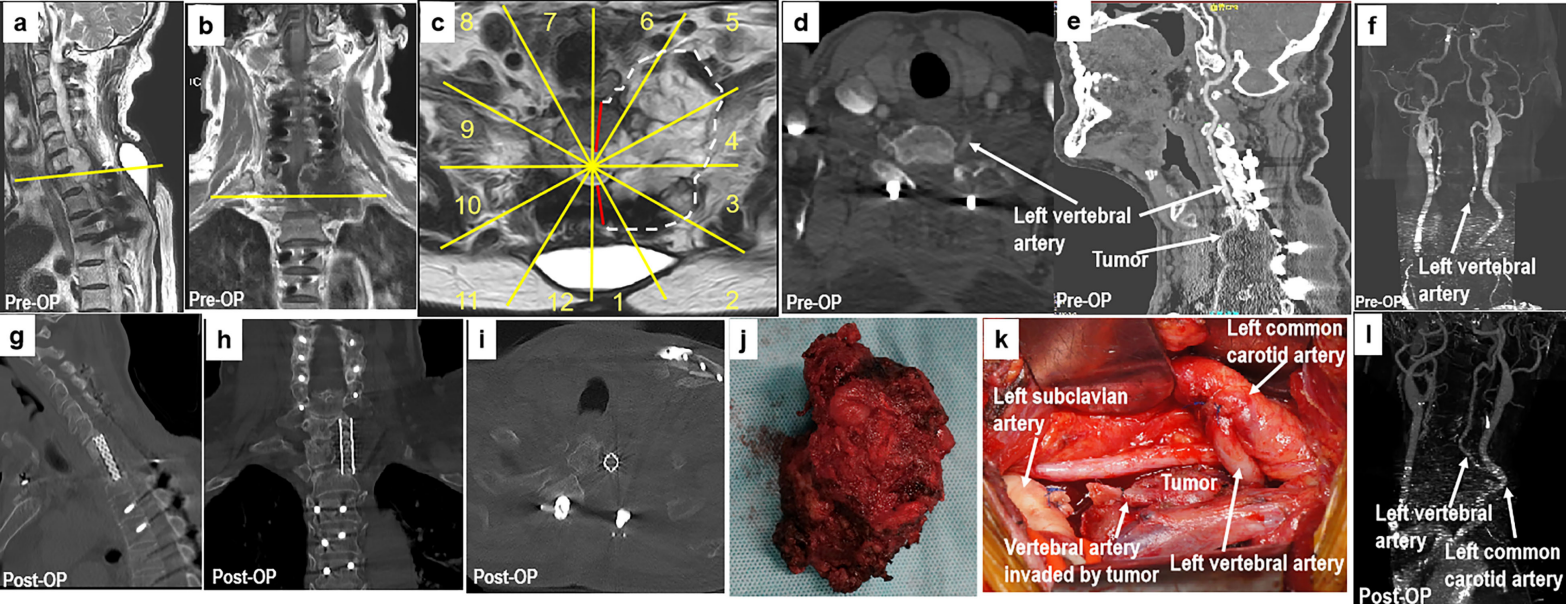
A 70-year-old man with chondrosarcoma at C7–T1. The patient had undergone a piecemeal resection of a tumor in the paravertebral region of the cervicothoracic junction at another hospital. Ten months later, the patient experienced numbness in his upper left arm. MR images **(A–C)** show tumor recurrence along the left side of C7 to T1 paravertebral CT scans **(D, E)**, and CT angiography **(F)** shows that the left vertebral artery was invaded by the tumor (sectors 1–5, **D, F**, WBB). Based on the WBB classification of the tumor, sagittal en bloc resection was performed using the rotation–reversion technique with a combined approach. The left vertebral artery was cut off outside the tumor and anastomosed with the left common carotid artery **(K)**. Postoperative CT angiography showing fluent blood flow in the left vertebral artery **(L)**. Postoperative CT scanning **(G–I)** and a photograph of the gross specimen **(J)** showing the margins of sagittal en bloc resection. WBB, Weinstein–Boriani–Biagini.

Eleven patients with primary spinal tumors who underwent en bloc resection using the rotation–reversion technique criteria were included. The demographic and clinical characteristics of these patients are summarized in **Tables 1**, **2**. **Figures 3**–**6** illustrate the en bloc resection techniques performed in four of these patients.

**Table 1:** Clinical characteristics of the patients included in this study.

No.	Age	Sex	Histology	Levels involved	Max-diameter, cm	Enneking stage	WBB classification	Previous surgery	Pre-OP chemo	Pre-OP radiation	Surgical approach	Staged surgery	Operation time, min	Blood loss, ml	Reconstruction method	Complications	Margin	Follow-up, months	LR	Oncological status	Interbody fusion, months
1	46	F	Malignant neurilemmoma	L3	8.1 × 6.8 × 9.1	IIB	7-2/A-D	−	+	−	Combined	−	1,060	4,500	AV/PS	Wound problem	Wide	42	−	NED	–
2	41	M	GCT	T9–T11	8.5 × 9.3 × 6.0	S3	2-8/A-D	−	−	−	Combined	−	485	4,000	EC/PS	Pneumonia, IF	Marginal	69	−	NED	32
3	61	M	Malignant neurilemmoma	L4–L5	9.1 × 7.7 × 19.5	IIB	1-6/A-D(SEBR)	−	+	−	Combined	−	980	4,000	TM/PS	IF	Marginal	27	−	DOD	–
4	36	M	Solitary fibroma	L5	5.6 × 7.9 × 16.9	S3	9-1/A-D	−	+	−	Combined	−	860	9,600	TM/PS	Wound problem	Marginal	35	−	NED	7
5	65	F	Malignant paraganglioma	T1–T3	8.2 × 6.8 × 8.4	IB	5-10/A-D	−	−	+	Combined	+	1,340	17,700	TM/PS	Wound problem, pneumonia	Marginal	29	−	NED	6
6	40	M	Osteosarcoma	T11–L2	8.8 × 9.4 × 11.9	IIB	5-1/A-D	+	+	+	Combined	−	1,180	12,200	TM/PS	Dura tear, CSFL, DVT	Intralesional	8	+	DOC	–
7	70	M	Chondrosarcoma	C7–T1	8.4 × 4.5 × 6.7	IIB	2-6/A-D, F(SEBR)	+	−	−	Combined	−	465	5,500	TM/PS	Pneumonia	Marginal	12	−	NED	5
8	34	F	Myxoid chondrosarcoma	T2–T5	14.2 × 10.8 × 11.4	IB	1-7/A-D	−	−	−	Posterior	−	880	5,500	TM/PS	Pneumonia	Marginal	28	−	DOD	–
9	22	F	GCT	T9–T11	6.0 × 6.3 × 5.0	S3	3-10/A-D	−	−	−	Posterior	−	560	5,200	AV/PS	Pneumonia	Marginal	36	−	NED	6
10	46	M	Osteosarcoma	L4–L5	6.1 × 6.3 × 5.5	IIB	7-11/A-D(SEBR)	+	+	−	Combined	−	1,050	6,000	TM/PS	Wound problem	Marginal	15	−	NED	7
11	25	M	GCT	T5–T7	6.0 × 7.2 × 6.2	S3	4-12/A-D	−	−	−	Posterior	−	480	2,500	AV/PS	Pneumonia	Marginal	8	−	NED	–

AV, artificial vertebrae; AWD, alive with disease; CSFL, cerebrospinal fluid leakage; Chemo, chemotherapy; DOC, died from other cause; DOD, died of disease; DVT, deep venous thrombosis; EC, expandable cage; F, female; GCT, giant cell tumor; IF, instrumentation failure; LR, local recurrence; M, male; NED, no evidence of disease; Pre-OP, preoperative; PS, pedicle screw; SEBR, sagittal en bloc resection; TM, titanium mesh; WBB, Weinstein–Boriani–Biagini.

**Table 2:** Epidemiologic, clinical, and surgical data.

Parameter	Value
Patients (n)	11 (4 F and 7 M)
Age, mean (range)	44.2 ± 15.6, (22–70) years
Diagnosis	
GCT	3
Malignant neurilemmoma	2
Osteosarcoma	2
Chondrosarcoma	2
Malignant paraganglioma	1
Solitary fibroma	1
Site	
CT	1
T	5
TL	1
L	4
Median size tumor	
Anteroposterior dimension (range)	8.1 ± 2.4 cm (5.6–14.2 cm)
Transverse dimension (range)	7.5 ± 1.8 cm (4.5–10.8 cm)
Axial dimension (range)	9.7 ± 4.8 cm (5.0–19.5 cm)
Enneking staging (no., [%])	
S3	4 (36.4%)
IB	2 (18.2%)
IIB	5 (45.5%)
WBB classification	
Sectors involved	
5 sectors	3
6 sectors	2
7 sectors	2
8 sectors	2
9 sectors	2
Layers involved	
A–D	10
A–D, F	1
Pre-OP treatment	
Chemotherapy	5
Radiation	2
Pre-OP neurologic status	
ASIA B	1
ASIA C	9
ASIA D	1
Parameter	Value
Post-OP neurologic status (last follow-up)	
ASIA B	1
ASIA D	8
ASIA E	2
Surgical approach (no., [%])	
Anterior-posterior	8 (72.3%)
Posterior	3 (27.3%)
Level of resection	
One-level	2
Two-level	3
Three-level	4
Four-level	2
Margin (no., [%])	
Marginal	9 (81.2%)
Wide	1 (9.1%)
Intralesional	1 (9.1%)
Operation time mean (Range)	849.1 ± 308.8 min (465–1,340 min)
Blood loss mean (Range)	6,972.7 ± 4,489.1 ml (2,500–17,700 ml)
Complications	
Pneumonia	6
Wound problem	4
IF	2
CSFL	1
Dura tear	1
DVT	1
Perioperative complication (no., [%])	10 (90.9%)
Major	6 (54.5%)
Minor	5 (45.5.0%)
Follow-up mean	28.1 ± 17.9 mons (8–69 months)
Local recurrence (no., [%])	1 (9.1%)
Metastases (no., [%])	3 (27.3%)
Dead (no., [%])	3 (27.3%)
Died from disease with evidence of LR at time of death	1 (9.1%)
Died from disease without evidence of LR at time of death	2 (18.2%)

CSFL, cerebrospinal fluid leakage; DVT, deep venous thrombosis; F, female; GCT, giant cell tumor; IF, instrumentation failure; LR, local recurrence; M, male; Pre-OP, preoperative; Post-OP, postoperative; WBB, Weinstein–Boriani–Biagini.

### 3.2 Oncology results

En bloc resection was achieved in all 11 patients. One wide margin (patient 1) was achieved with the epidural extension of the tumor fully contained by the ligamentum flavum in the resected specimen (**Figure 3**). Marginal margins were obtained at the tumor capsule along the dura in nine patients. One patient (patient 6) had an intralesional margin due to intracanal tumor contamination following initial surgery (**Figure 5**).

Perioperative complications were classified as major or minor (19). Any complication that substantially alters an otherwise smooth and expected course of recovery was considered a major complication; others were defined as minor. Six patients (55%) experienced major complications, and five (45%) experienced minor complications, with 10 (91%) of the 11 patients experiencing a perioperative complication. One patient (patient 6) experienced local recurrence 3 months after surgery; this patient, who had T11-L2 osteosarcoma with intralesional margins due to contamination following previous operations, died of deep vein thrombosis 8 months after surgery. Patients 3 and 8 died of lung metastases at 27 and 28 months, respectively, after surgery without local recurrence.

### 3.3 Functional results

All 11 patients had different degrees of postoperative neurological deficits due to the resection of nerve roots of the diseased vertebra(e). No postoperative ischemic spinal cord injury or delayed neurologic deficit occurred. At the last follow-up after rehabilitation, two patients were able to normally walk (Frankel E), and eight were able to walk with crutches (Frankel D). One patient (patient 8) with complete paralysis (Frankel B) preoperatively did not experience significant improvement in motor function. Interbody fusion was confirmed by CT scans in six (55%) of the 11 patients. Instrumentation failures were observed in two patients (patients 2 and 3), both of whom underwent revision surgery while normal spinal alignment was maintained.

## 4 Discussion

### 4.1 Background and rationale

A system for the surgical staging of primary musculoskeletal tumors, first proposed by Enneking in 1980, elucidated the principles of tumor excision and the concept of surgical margins based on other types of oncological surgery (18). En bloc resection is performed to remove the tumor in a single piece with tumor-free margins and without any tumor contamination (20). Wide or radical margin en bloc resection was recommended for the removal of primary malignant tumors (18). The WBB classification was later developed in order to apply these principles of tumor resection to the removal of spinal tumors (17). The WBB classification system also provided a common terminology for describing the details of the tumor and evaluating the results of treatment for spinal tumors (17).

Regional anatomical constraints and important tissue structures surrounding the spine restrict the application of the Enneking system in the resection of spinal tumors (6–8, 11). Resection with tumor-free margins can seldom be achieved in huge tumors originating from the spine, because of the complex anatomy, large tumor volumes, and invasion of important structures (21, 22). The proposed approaches consist usually of the posterior approach alone, or combined anterior and posterior approaches for separation of the anterior structures involved (23, 24). Frequently, the dura sac and nerve root cannot be easily released from the lesion without direct visualization, as the lamina and pedicle invasion made lesion removal difficult because of its connection with the sac through the nerve root(s), limiting the ability to obtain safe margins along the dura. The rotation–reversion technique was developed and utilized in the en bloc resection of huge primary tumors with tumor invasion that included not only the vertebral body but also the pedicle and part of the posterior arch in the mobile spine.

### 4.2 Can the rotation–reversion technique achieve oncologically appropriate margins in en bloc resection of huge primary tumors with epidural involvement in the mobile spine?

The WBB classification system has defined the margins of extraosseous and intraosseous lesions of the spine, and this stage was set on the premise that the spinal cord can not be resected. Without dural resection, marginal en bloc resection may be the best margin at the tumor capsule along the dura, obtained in most of the spinal tumors with epidural extension. If the epidural layer is only displaced anteriorly by the tumor, but without infiltration (layer D), surgical dissection may involve separating the intact tumor capsule from the dura surface. If the epidural extension of the tumor is fully contained by natural barriers, such as the ligamentum flavum or longitudinal ligament, on the resected specimen, the margins along the dura could be considered wide (24). If there is intradural involvement (layer E), the infiltrated dura should be removed entirely with the specimen to obtain safe margins (11, 12). The rotation–reversion technique provides direct visualization of the ventral and dorsal structures of the spinal column, allowing the tumor to be sectioned with safe margins and confirming that the margins along the dura are not contaminated. Of the 11 patients in this study, 10 (91%) had negative margins, including one with wide and nine with marginal margins. Similarly, a systematic review reported that the negative margin rate obtained following en bloc resection of 229 primary spinal tumors was 86.1% (25), and a study from a single center of 220 spinal tumors that underwent en bloc resection found that the negative margin rate was 85.9% (23). Careful planning and efficient design of surgical procedures can be successful in obtaining oncologically appropriate margins, even in difficult cases (9).

### 4.3 What are the complications of the rotation–reversion technique?

The mean operation time in this study was 849.1 min, and the mean blood loss was 6,972.7 ml. In comparison, a study of en bloc resection of 22 spinal tumors reported a shorter mean operation time (493 min; range 150–840 min) and a lower volume of blood loss (1,895 ml; range 150–6,500 ml) (26). These findings cannot be readily compared because of differences in study populations and the small numbers of patients in each study. Tumor volume was significantly greater in the present study, with all tumors having expanded into the canal with extradural extension. These factors made surgical manipulations in the present study more difficult and time-consuming, resulting in higher intraoperative blood volumes. A systematic review and meta-analysis of 44 articles that included 306 patients who underwent en bloc resection for spinal tumors found that the mean operation time was 12.1 h (range 2–42 h) and that the median blood loss was 3.7 L (range 0.1–37 L) (25). More recently, another patient-level meta-analysis including 582 patients who underwent en bloc spondylectomy reported a median operation time of 555 min and a median blood loss of 2,000 ml (27).

Morbidities of en bloc resection of spinal tumors are thought to be worse than morbidities associated with the resection of other tumors because en bloc resection of spinal tumors is more technically demanding (10). The rate of perioperative complications in the present study (91%) was higher than rates reported previously (35% to 65%) (10, 26, 28), potentially because patients in the present study required more difficult manipulations of neurovascular structures due to the huge volumes of these tumors and the involvement of the dura with nerve roots. However, none of the patients in the present study died intraoperatively or experienced other fatal complications. In comparison, the mortality rate of patients who have undergone en bloc resection of spinal tumors was reported to range from 0% to 7.7% (22, 29, 30). The most frequent major complication observed in the present study was wound healing problems, occurring in four (36%) of the 11 patients. Wound healing problems may be caused by the need to excise large amounts of soft tissue and vascular structures to obtain safe margins due to the huge volume of the tumors, which may result in insufficient coverage and reduced blood supply to the wound. Pneumonia was the most frequent minor complication, occurring in six patients. Pneumonia may be related to atelectasis, which is caused by pleural rupture during an operation. None of the patients in the present study experienced injuries to important vessels or the spinal cord during surgery. The rotation–reversion technique allowed careful separation of the surrounding neurovascular structures from the tumor under direct vision, thereby avoiding serious complications caused by intraoperative rough manipulations.

### 4.4 How does the rotation–reversion technique alter oncologic and functional outcomes after tumor resection?

Only one (9%) of the 11 patients died of disease with evidence of local recurrence at the time of death. None of the other 10 patients experienced recurrence throughout follow-up. Reconstruction of the integrity of the spinal column after en bloc resection is critical for optimal postoperative functional results. Spinal reconstruction is regarded as particularly challenging when tumors are huge and involve multiple vertebral levels, as more vertebral bone and surrounding structures must be resected totally to excise the entire tumor mass. The instrument failure rate after en bloc resection of spinal tumors has been found to range from 7% to 40% (10, 22, 31), in agreement with the instrument failure rate in the present study, 18.2%. Interbody fusion was confirmed in six (55%) patients in the present study. The lower fusion rate in the present study may be due to the diminished vitality and poor healing capacity of the bone, resulting from the extensive intraoperative vascular dissection required to separate the huge masses.

### 4.5 Limitations

One important limitation of the present study was the small sample number, as this study included only 11 patients. In addition, patients with extraosseous soft tissues and intraosseous tumors without dural involvement were not included. Furthermore, the results of the present study could not be compared with the results of other studies of en bloc resection, due to wide variations in patient and tumor parameters and the relatively short follow-up time. Additional studies with larger numbers of patients, longer follow-up times, and control groups are needed to establish a definitive role of the rotation–reversion technique in the en bloc resection of spinal tumors.

## 5 Conclusions

The rotation–reversion technique based on the WBB classification is a feasible, safe, and effective procedure for en bloc resection of large primary spinal tumors that extend not only into the vertebral body but also into the pedicle and part of the posterior arch. This procedure is reliable in achieving safe oncological margins with satisfactory local control in selected patients.

## Data availability statement

The original contributions presented in the study are included in the article/supplementary material. Further inquiries can be directed to the corresponding author.

## Ethics statement

The studies involving human participants were reviewed and approved by the ethics committee of The Third Affiliated Hospital of Southern Medical University. Written informed consent to participate in this study was provided by the participants’ legal guardian/next of kin. Written informed consent was obtained from the individual(s), and minor(s)’ legal guardian/next of kin, for the publication of any potentially identifiable images or data included in this article.

## Author contributions

ML: writing—original draft, data curation, and funding acquisition. ZZ: investigation and methodology. WC: formal analysis and software. ZL: validation and project administration. SWD: resources and visualization. CH: formal analysis and investigation. HL: conceptualization, funding acquisition, project administration, supervision, and writing—review and editing. DJ: supervision. SB: writing—review and editing. All authors contributed to the article and approved the submitted version.

## Funding

This work was supported financially by the Research and Development Projects in Key Areas of Guangdong Province (No. 2019B020201015) and the Natural Science Foundation of Guangdong Province (No. 2021A1515011313).

## Acknowledgments

The authors thank Dr. Yuheng Lu for his excellent drawings of our surgical plans.

## Conflict of interest

The authors declare that the research was conducted in the absence of any commercial or financial relationships that could be construed as a potential conflict of interest.

## Publisher’s note

All claims expressed in this article are solely those of the authors and do not necessarily represent those of their affiliated organizations, or those of the publisher, the editors and the reviewers. Any product that may be evaluated in this article, or claim that may be made by its manufacturer, is not guaranteed or endorsed by the publisher.

## Author disclaimer

The content is solely the responsibility of the authors and does not necessarily represent the official views of the funders.
